# Internet use, employment performance and the health of Chinese residents

**DOI:** 10.1093/inthealth/ihab034

**Published:** 2021-06-15

**Authors:** Kewen Yang

**Affiliations:** College of Economics and Management, Northwest A&F University, No. 3 Taicheng Road, Yangling, Shaanxi, China, 712100

**Keywords:** employment performance, health, Internet, instrumental variable

## Abstract

Using data from the China Labor-force Dynamics Survey 2016, this study examines the effects of Internet use on Chinese resident health and the role of employment performance in this process. The results show that Internet use had a significant positive effect on the health of Chinese residents. After addressing potential endogeneity by applying appropriate instrumental variable estimation, the main findings remain robust. Heterogeneity analysis found that the Internet is conducive to improving the health of older, less-educated and female residents, which suggests that the Internet can narrow the health gap of residents with different ages, education levels and genders. Through the analysis of the impact mechanism, it was found that although Chinese residents can improve their health by enhancing their employment performance with the help of the Internet, the mediating effect accounts for <20%, indicating that the role of employment performance in the process of improving the health of Chinese residents through Internet use is very limited. Finally, suggestions are given to promote the healthy development of China via the effects of Internet use.

## Introduction

Since the reform and opening up, the mode of extensive economic growth relying on resource investment has led to the rapid development of China's economy to become the second largest economy in the world in 2010. However, with the continuous progress of China's industrialization, extensive economic growth is increasingly difficult to sustain. In the future, improving the contribution of total factor productivity to economic growth will be key to China's successful industrialization. In this context, the importance of human capital is increasing.^1^ Health is not only an important component of human capital, but also an important goal of economic development.^2^ To this end, the Chinese government continues to implement medical and health system reforms in an attempt to solve the health problems of residents in a more effective manner.

However, according to Grossman's^3^ theory of health capital demand, health is the result of multiple factors. Although medical and health services play important roles in treating diseases and improving health, because of the corresponding high medical expenditure, residents can easily fall into ‘poverty due to illness’ and ‘poverty back due to illness’. Therefore, finding a way to improve the health of residents at a lower cost is a major goal of health economics.

With the proposal of the ‘Broadband China’ strategy, China's Internet industry is developing rapidly. According to the 44th Statistical Report on Internet Development in China (http://www.cac.gov.cn/2019-08/30/c_1124938750.htm), by June 2019, the number of Internet users in China had reached 854 million, with an Internet penetration rate of 61.2%. According to the World Internet Development Report 2018, China's Internet Development Index ranks second in the world, second only to the USA. Against this background, in 2015, the State Council's guiding opinions on actively promoting the ‘Internet +’ action noted that China should accelerate the development of ‘Internet +’ and enable Internet innovation to be deeply integrated with all sectors of the economy and society, giving full play to the important role of ‘Internet +’ in promoting the healthy development of the economy.

The combination of the Internet, medical treatment and health makes it convenient for residents to receive medical services and obtain health information to prevent diseases. A lack of professional medical knowledge leads to information asymmetry between doctors and patients, which enables the ‘induced demand’ of medical services.^4^ Therefore, trust between doctors and patients has become an important factor hindering treatment.^5^ The rapid development and popularization of the Internet has greatly reduced the cost of information dissemination and provided a new means for the diffusion and acquisition of medical information, which is conducive to reducing the induced demand and improving the trust between doctors and patients to improve the medical effect. Moreover, because of people's attention to health problems, the health information provided by the Internet is increasingly rich, thereby providing a possible new means of disease prevention and healthcare.

With the advancement of industrialization, employment performance and residents’ health are increasingly related. According to Grossman's^3^ theory of health capital demand, income and environment are important factors affecting residents’ health. Employment performance is closely related to residents’ income and working environment. However, information asymmetry and incompleteness often exist in the labour market, which makes it difficult to achieve effective matching between supply and demand. The communication and big data analysis functions of the Internet are conducive to breaking down information barriers, promoting effective matching between supply and demand and improving employment performance.^6^

Therefore, there are important questions to consider. Can Internet use improve people's health? What role does employment performance play? This article uses China Labor-force Dynamics Survey (CLDS) data from 2016 to investigate the above issues. Compared with the existing literature, the marginal contributions of this article are as follows. First, few studies consider the impact of Internet use on residents’ health from the perspective of information technology and the results of this article provide a deeper understanding of the relationship between Internet use and residents’ health. Second, from the perspective of employment performance, this article examines the possible channels through which the Internet may affect residents’ health. On the one hand, this article overcomes the shortcomings of existing research; on the other hand, it provides a practical basis for guiding Internet use to improve residents’ health. Third, the study of the relationship between Internet use and residents’ health is conducive to the evaluation of the value and significance of China's Internet development from the perspective of health. Moreover, this information can provide effective policy suggestions for China's Internet development from the perspective of health and promote the healthy development of China's economy and society.

## Literature review

Whether to invest in health depends on the benefits and costs of the investment. If Internet use can cause residents to obtain higher incomes and better jobs, these improvements will increase benefits and reduce costs of investment in health. If other conditions remain unchanged, it is beneficial to increase health investment to improve health.^3^

Under this assumption, this article focuses on three aspects of the literature: the relationship between Internet use and health, whether increased employment performance can improve health and whether Internet use can improve employment performance.

### Internet use and health

There is no consistent conclusion about the relationship between Internet use and health. Some studies have shown that Internet use helps the elderly to communicate with others with similar hobbies and interests, which improves social support and reduces loneliness, thereby improving health, especially for the elderly with health problems or mobility difficulties.^7–11^ For example, Jin and Zhao^12^ used 2016 China Longitudinal Aging social data to investigate the impact of Internet use on the health, social participation and life satisfaction of the elderly: the results show that Internet use can improve health, social adaptation and life satisfaction. Other studies suggest that the Internet also provides residents with variety of entertainment methods,^6^ which can lead to addiction and health deterioration.^13–15^ For example, Billari et al.^16^ used 2008, 2010 and 2012 German Socio-Economic Panel (SOEP) data to investigate the impact of Internet use before bed on sleep: the results show that Internet use led to sleep deprivation and decreased sleep satisfaction; moreover, sleep disorders tend to cause depression and harm health.^17^

### Employment performance and health

Job characteristics are important factors affecting health. Various forms of capital, such as educational, health and financial capital, can transform each other in terms of individuals obtaining income and completing tasks. When financial capital and educational capital are low, it is often necessary to rely on health capital to obtain income,^18^ which makes job characteristics an important factor affecting health.^19–23^ First, the labour force engaged in higher positions may have more health knowledge themselves or be able to more easily access doctors and other personnel with more health knowledge, which is conducive to improving the utilization efficiency of medical resources. Second, jobs with higher positions and self-employment have greater flexibility. Such employees and employers can arrange their working hours according to their own needs, which is conducive to timely medical treatment and taking care of others.^24^ Finally, more senior jobs tend to be less likely to be exposed to risk factors, reducing the damage to health caused by the work environment.^2^

The impact of employment performance on health is reflected not only in job characteristics, but also in income.^23^ First, higher income leads to a more balanced and reasonable diet, which helps to prevent obesity, hypertension and other related diseases.^25^ Second, higher income is conducive to special health investment, such as tourism, vacation and fitness exercise, among others, which helps to improve the physical and mental health of residents.^26^ Third, higher income can lead to regular physical examination, timely detection of health problems and medical treatment, improving the possibility of curing diseases.^2^

### Internet use and employment performance

Internet use has an important impact on employment performance. As early as the beginning of Internet development, Autor^27^ proposed that Internet use may have an important impact on the labour market in the following three respects. First, Internet use can improve the match between labour and business. Information asymmetry and incomplete information widely exist in the labour market. Internet use can provide information at a lower cost to help enterprises and the labour force understand each other and realize effective matching. Second, Internet use benefits the efficient delivery of labour services. The Internet enables the rapid transfer of information, which allows some work to be done outside the enterprise. For the labour force engaged in these jobs, working at home can not only save time spent in traffic, thus providing more effective services, but also balance the relationship between family and work and reduce retention wages. Finally, Internet use can affect the demand of enterprises for the labour force. Limited by geographical distance, traditional enterprises have difficulty achieving economies of scale. With the information cost advantage of the Internet, e-commerce enterprises can expand their market scale at lower cost, which promotes specialization, expands labour demand and increases employment.

On the basis of the above analysis, various scholars have investigated the relationship between these factors, although some scholars have not found that Internet use promotes employment.^28,29^ However, over time, the employment effect of Internet use has consistently emerged.^30–32^ For example, Dettling^6^ investigated the impact of Internet use on labour supply using data from the US Census current Population Survey (CPS) from 2000 to 2009. The results showed that while Internet use had no effect on male and single women's labour participation, it increased the labour supply of married women, and the effect was greater among married women with higher education and children. A supplementary analysis found that Internet use helped increase employment by encouraging married women to take more flexible jobs and save time in home production.

According to the above literature review, Internet use has an important impact on health, and employment performance may be an important mechanism through which Internet use influences health. Empirical tests of these factors will be carried out later.

## Research design

### Model specification

This article examines the relationship between Internet use and the health of Chinese residents. Considering that the indicators used to measure health include continuous variables, dichotomized variables and five-category variables, the basic econometric models in this article can be divided into three categories. First, for continuous variables, the ordinary least squares model is defined as follows:

(1) \begin{equation*}{Y_i} = \alpha + \beta Interne{t_i} + \gamma {X_i} + {\mu _c} + {u_i}.\end{equation*}

Second, for dichotomous variables, the following probit model is used:

(2) \begin{equation*}\Pr \!({Y_i} = 1) = \Phi (\alpha + \beta Interne{t_i} + \gamma {X_i} + {\mu _c} + u_i).\end{equation*}

Third, for five-category variables, this article implements the following ordered probit model:

Assume that the range of the original value of a health variable is 1 . . . , m; the ordered probit model can be expressed as

(3) \begin{equation*}{Y_i} = j,\quad {\rm{if}}\,\,{u_{j - 1}} < Y_i^* < = {u_j},\quad j = 1, \ldots ,m,\end{equation*}

where $Y_i^*$ is a latent continuous variable behind the ordered categorical variable *Y_i_* and is affected by *Internet_i_*, demographic and socio-economic variable *X_i_* and regional characteristics *μ_c_*:

(4) \begin{equation*}Y_i^* = \beta Interne{t_i} + \gamma {X_i} + {\mu _c} + {u_i},\quad {u_i}\sim N(0,1).\end{equation*}

In addition, *u*_0_ = −∞, *u_j_*≤*u_j_*_+1_, *u_m_* = ∞. According to the assumption of *u_i_* in equation (4), the probability that *Y_i_* takes a value of *j* is

(5) \begin{eqnarray*}{P_{ij}} &=& P({Y_i} = j) = \Phi ({u_j} - \beta Interne{t_i} - \gamma {X_i} + {\mu _c})\nonumber\\ && -\,\, \Phi ({u_{j - 1}} - \beta Interne{t_i} - \gamma {X_i} + {\mu _c}),\end{eqnarray*}

where Φ(·) is the cumulative density function of the standard normal distribution and *j* = 1–5. If *β* is positive, then as the explanatory variable increases, the probability of low-level values decreases and the probability of high-level values increases.

In these equations, *i* represents a resident and *Y_i_* represents the health of resident *i. Internet_i_* represents Internet use, *X_i_* represents the control variable, *μ_c_* represents the city's fixed effect, *u_i_* is a random disturbance term and *β* and *γ* represent the coefficients of the corresponding variables, respectively.

The above basic model may produce biased and inconsistent estimates due to problems of endogeneity.^8,16^ The endogeneity results from two main aspects. The first is the problem of omitted variables. For example, higher-capacity residents not only have better Internet use skills,^16^ but also generally have better health due to lower investment costs.^33^ The second is reversed causality: poor health may reduce the likelihood of using the Internet.^8^ In summary, we believe that the basic model may suffer from endogeneity. Therefore this article uses instrumental variables to address this issue, as explained below in detail.

### Data and variables

This article uses 2016 CLDS data. The data adopt the scientific probability sampling and rotation sample tracking to adapt to China's rapidly changing environment and to account for the characteristics of cross-sectional surveys. The CLDS has established a comprehensive database of the 15- to 64-year-old labour force (including those ≥65 y of age who are still working) as the survey object, including three levels of tracking and cross-sectional data of individuals in the labour force, family and community. The 2016 CLDS covers a nationally representative sample of 29 provinces and cities in China, with a sample size of 401 village houses, 14 226 households and 21 086 individuals. During data collation, individuals >64 y and <15 y, individuals who are still in school and individuals with missing key variables such as age are removed: the final sample size is 12 199.

The dependent variable is health. Health indicators can be roughly divided into objective and subjective indicators. Subjective indicators, though simple, have been shown to be predictive of mortality and disability.^34^ In addition, subjective health indicators are more in line with the definition of health provided by the World Health Organization (WHO). This definition of health considers not only the absence of disease and good physical fitness, but also physical and psychological health and social welfare.^35^ To more fully examine the impact of Internet use on the health of residents in China, this article uses both subjective and objective health indicators.

The following subjective indicators were used: (1) self-rated health based on the question ‘What do you think of your current health status?’, with answers ‘very unhealthy’, ‘relatively unhealthy’, ‘fair’, ‘relatively healthy’ and ‘very healthy’ represented by 1–5, respectively; (2) physical pain, measured by the question ‘Have you experienced any physical pain in the past month?’ and the answers ‘always’, ‘often’, ‘sometimes’, ‘rarely’ and ‘none’ represented by 1–5, respectively; and (3) depression, which is measured by the question ‘How often did the following situation occurred last week’ for 20 minor issues, such as ‘worrying about small things’, ‘not wanting to eat’, ‘feeling depressed’, ‘struggling to do anything’, ‘feeling scared’ and ‘not sleeping well’. The answers are ‘no or almost none’, ‘rare’, ‘often’ and ‘almost always’. This article assigns ‘no or almost none’ to 4, ‘rare’ to 3, ‘usual’ to 2 and ‘almost always’ to 1 and then adds the scores for all 20 questions to obtain the total value. Therefore a larger value indicates a lower level of depression.

Indicators for measuring objective health include a question about injuries: ‘Are there any injuries in the past two weeks?’ The possible answers are no (1) and yes (0).

The main explanatory variable is Internet usage. Based on the availability of data and reference to the research by Dettling,^6^ this article uses the question ‘How has your family used the Internet in the past year?’ to measure Internet use. The answers to this question are ‘Only use a computer to access the Internet (including tablets)’, ‘use only mobile phones to access the Internet’, ‘use both computers and mobile phones to access the Internet’ and ‘no Internet’. This article classifies ‘no Internet’ as a unique category and assigns a value of 0; the other answers are combined into the same category and assigned a value of 1.

In addition, referring to health capital theory and existing research,^3,8,9^ this article controls for individual demographic and social characteristics, such as gender, age, residential location, education, marital status, and medical insurance; family characteristics, such as family social hierarchy 5 y ago and toilet type; individual health behaviour characteristics, such as smoking and drinking history and sports participation; and community characteristics, such as the degree of air pollution [According to the reviewer's suggestion, improper variable control may lead to unreasonable results. For this reason, this article excludes some control variables that may give rise to selection bias (such as medical insurance and individual health behaviour characteristics) and the results are still robust, which shows that the selection of control variables in this article is reasonable. Due to the limited space, the results cannot be presented and can be obtained from the author if necessary.] Taking into account the differences in socio-economic development levels and customs in different regions, this article further controls for the fixed effects of cities. More than one member of a sampled family that meets the age requirements may be a participant in the labour force, so this article clusters at the family level. See Table 1 for the processing results, definitions of the variables and descriptive statistical results.

**Table 1.** Descriptive statistics of major variables

Variables	Meaning/value	Observations	Mean	Standard deviation	Minimum	Maximum
Dependent variables						
Self-rated health	Five-category variable	12 199	3.687	0.954	1	5
Physical pain	Five-category variable	12 199	4.056	1.119	1	5
Depression	Continuous variable	12 199	73.06	8.528	20	80
Injury	1, no; 0, yes	12 199	0.903	0.296	0	1
Explanatory variables						
Internet usage	1, yes; 0, no	12 199	0.676	0.468	0	1
Individual characteristics						
Sex	1, male; 0, female	12 199	0.530	0.499	0	1
Age	Years	12 199	44.09	11.60	15	64
Residential location	1, urban; 0, rural	12 199	0.351	0.477	0	1
Education	Years	12 199	9.016	3.985	1	22
Marital status	1, married; 0, not married	12 199	0.870	0.336	0	1
Medical insurance	1, yes; 0, no	12 199	0.918	0.275	0	1
Family characteristics						
Social hierarchy	Higher values indicate higher levels	12 199	3.849	1.770	1	10
Indoor independent toilet	1, yes; 0, no	12 199	0.611	0.488	0	1
Individual health behaviour						
Smoking history	1, yes; 0, no	12 199	0.320	0.466	0	1
Drinking history	Same as above	12 199	0.231	0.422	0	1
Sports	Same as above	12 199	0.283	0.451	0	1
Community characteristics						
Air pollution	1, yes; 0, no	12 199	0.203	0.403	0	1

Data source: 2016 CLDS.

Overall, Chinese residents have good health, with high subjective and objective health indexes, on average, and the worst self-rated health index is 3.7, which is between average and healthy. The results of the above health indicators are relatively consistent, indicating that the measurement of residents’ health status is relatively robust. China's household Internet penetration rate is approximately 67.6%.

## Estimation results and analysis

### Benchmark regression

As seen in equations (1)–(5), this article uses a variety of econometric models for estimation; the results are shown in Tables 2 and 3.

**Table 2.** Basic model

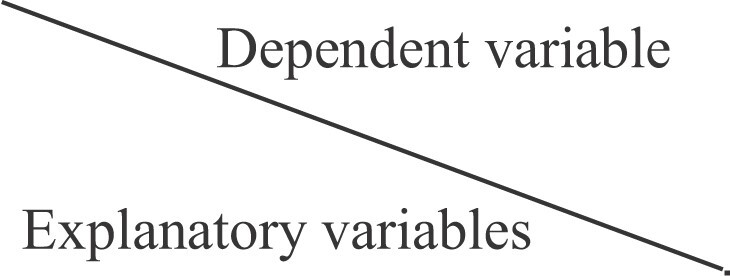				
	1	2	3	4
	Self-rated health	Physical pain	Depression	Injury
Main explanatory variables				
Internet usage	0.138^***^	0.063^**^	0.644^***^	0.013^*^
	(0.029)	(0.029)	(0.232)	(0.008)
Individual characteristics				
Male	0.127^***^	0.226^***^	1.773^***^	0.107^**^
	(0.026)	(0.028)	(0.193)	(0.045)
Age	−0.027^***^	−0.020^***^	−0.033^***^	−0.007^***^
	(0.001)	(0.001)	(0.009)	(0.002)
Urban	0.022	0.093^**^	−0.024	−0.018
	(0.037)	(0.039)	(0.270)	(0.059)
Education	0.009^**^	0.017^***^	0.120^***^	0.009
	(0.004)	(0.004)	(0.028)	(0.006)
Married	0.114^***^	0.093^***^	1.301^***^	0.147^***^
	(0.033)	(0.033)	(0.264)	(0.052)
Medical insurance	0.001	−0.003	0.229	−0.134^**^
	(0.038)	(0.040)	(0.305)	(0.064)
Family characteristics				
Social hierarchy	0.072^***^	0.056^***^	0.486^***^	0.047^***^
	(0.006)	(0.007)	(0.050)	(0.010)
Indoor independent toilet	0.066^*^	0.008	0.565^**^	0.065
	(0.035)	(0.035)	(0.263)	(0.054)
Individual health behavior				
Smoking history	0.037	0.015	−0.105	0.039
	(0.028)	(0.030)	(0.199)	(0.048)
Drinking history	0.064^**^	0.017	−0.120	0.127^***^
	(0.027)	(0.029)	(0.197)	(0.048)
Sports	0.083^***^	0.007	0.452^**^	−0.024
	(0.025)	(0.026)	(0.181)	(0.040)
Community characteristics				
Air pollution	−0.143^***^	−0.131^***^	−1.182^***^	−0.076^*^
	(0.028)	(0.029)	(0.217)	(0.044)
Constant	–	–	68.619^***^	1.194^***^
	–	–	(0.978)	(0.198)
City FE	Yes	Yes	Yes	Yes
Observations	12 199	12 199	12 199	12 199
Wald/F statistic	2650.96	1651.82	8.410	503.52
Pseudo-R^2^/adjusted R^2^	0.086	0.054	0.088	0.072

Values in parentheses are the cluster robust standard error. *p<0.1, **p<0.05, ***p<0.01. Due to space limitations, the cut point is omitted. In the probit model, the coefficient of Internet use represents the marginal effect.

**Table 3.** Marginal effect in the ordered probit model

Explanatory variables	1	2	3	4	5
	Self-rated health				
Internet usage	−0.003^***^	−0.021^***^	−0.023^***^	0.012^***^	0.035^***^
	(0.001)	(0.005)	(0.005)	(0.004)	(0.008)
	Physical pain				
	−0.002^*^	−0.010^**^	−0.010^***^	−0.002^*^	0.023^***^
	(0.001)	(0.005)	(0.004)	(0.001)	(0.011)

Values in parentheses are the Delta-method standard errors. *p<0.1, **p<0.05, ***p<0.01.

As shown in Table 2, Internet use has a significant impact on residents’ health. Compared with not using the Internet, Internet use increased self-rated health by 0.138 units in the probit model, increased physical pain by 0.063 units in the probit model, increased depression by approximately 0.644 and decreased the likelihood of suffering from injury by 1.3%. These results suggest that Internet use has a significant positive impact on residents’ health, which is consistent with the existing research results.^8,9^

Since self-rated health and physical pain are both five-category variables, the estimated coefficients in Table 2 reflect only the relative impact of Internet use, not the marginal effect. Therefore the marginal effects of Internet use on self-rated health and physical pain are examined in combination with the estimates of each cut-off point. The results are shown in Table 3. Internet use decreases the probability of classifying self-rated health as very unhealthy, relatively unhealthy and fair by 0.3%, 2.1% and 2.3%, respectively, and increases the probability of being classified as relatively healthy and very healthy by 1.2% and 3.5%, respectively. Moreover, Internet use decreased the probability of having physical pain always, often, sometimes and rarely by 0.2%, 1.0%, 1.0% and 0.2%, respectively, and increased the probability of having no pain by 2.3%.

According to the regression results, the control variables in Table 2 essentially match the theoretical expectations. In terms of individual characteristics, men are healthier than women.^36^ Furthermore, older individuals have worse physiological function.^37^ Additionally, residents living in cities and towns are healthier than individuals living in rural areas. Education improves investment efficiency and thus health.^37^ Moreover, married residents are healthier than unmarried residents.^8^ Medical insurance has a negative effect on injuries but does not affect subjective indicators such as self-rated health; this result may be due to adverse selection.

From the perspective of family characteristics, the higher the social hierarchy of the family is, the richer the available resources are, which can enhance individual health.^38,39^ Indoor toilets are more sanitary than outdoor public toilets and are conducive to improving health.^40^ From the perspective of individual health behaviour, drinking is found to have a positive impact on health, although previous studies have shown that drinking has a negative impact on health.^41^ However, studies have also shown that older people with a higher education are more likely to drink alcohol regularly.^42^ If we view drinking as a kind of preference, moderate drinking not only has little negative effect on health, but can also make people happy in body and mind and improve their health because of the effective satisfaction of individual needs. Clearly the data used in this article are rough, which may also affect the authenticity of the results. Therefore the relationship between drinking and health needs further study. Moderate exercise can also enhance health.^43^ From the perspective of community characteristics, the more serious the air pollution is, the greater the damage to the health of residents.^44^

#### Two-stage least squares (2SLS) method

The use of different health variables showed that Internet use could enhance the health of residents, but endogeneity could not be ruled out. Therefore this article made additional effort to select control variables. To reduce the problem of omitted variables caused by unobservable factors, it is necessary to control for the relevant influencing factors, such as the sociological characteristics of the individual population and health behaviours, family background, community environment and city fixed effects. Furthermore, the article searches for instrumental variables of residents’ Internet use to reduce the bias and inconsistency caused by possible endogeneity.

An effective instrumental variable must satisfy two conditions: it is not related to the random disturbance term and it is related to the endogenous variable. In this article, the average Internet usage of the community where the individual lives (excluding the Internet usage of the household itself) is used as an instrumental variable of the Internet usage of an individual household.

People living in the same community tend to show similar behaviour.^45^ In general, to save costs and obtain higher benefits, the network infrastructure has regional characteristics.^6^ If the network infrastructure is well developed in an area, the households in the community can easily access the network at any time. Therefore we expect that the interaction effect of behaviour will cause the Internet usage of households within a community to be related to the average level of usage in the community. The results are shown in Table 4. Regardless of whether other variables and regional fixed effects are controlled for, the average Internet use of the community has a significant positive correlation with the Internet use of individual households. That is, the instrumental variables are significantly correlated with endogenous variables, so the correlation hypothesis of instrumental variables is satisfied. In addition, regardless of whether other variables and regional fixed effects are controlled for, the F statistic value is >10, indicating that there is no weak instrumental variable problem.^46^

**Table 4.** Instrumental variable test correlation test

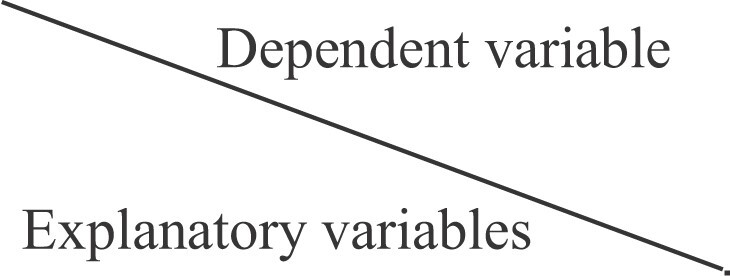				
	Internet usage			
	1	2	3	4
Average Internet usage of the community	1.021^***^	0.906^***^	0.747^***^	0.593^***^
	(0.014)	(0.031)	(0.022)	(0.036)
Control variables	No	No	Yes	Yes
City FE	No	Yes	No	Yes
Observations	12 199	12 199	12 199	12 199
F statistic	5238.16	218.74	763.21	97.23
Adjusted R^2^	0.363	0.372	0.430	0.442

Values in parentheses are the cluster robust standard errors. *p<0.1, **p<0.05, ***p<0.01. To save space, control variables and constants are not reported here. The control variables are the same as in Table 2.

Furthermore, whether the network infrastructure is well developed in a region is greatly influenced by the economic level and environment of the region. Therefore the average community Internet usage may be related to the economic level and environmental factors of the region and may in turn affect the health of residents in the community. Controlling for the city fixed effects and air pollution, average Internet use in a community is not related to the health of residents at the micro individual level. The design of this instrumental variable has previously been adopted by researchers.^6,47^

In view of the fact that there is only one instrumental variable, which has the characteristics of just identification, in order to test the exogenous of instrumental variables, this article uses the test method of Conley et al.^48^ for reference to test the exogenous of instrumental variables, and the results are shown in Table 5.

**Table 5.** Instrumental variable test: exogeneity test

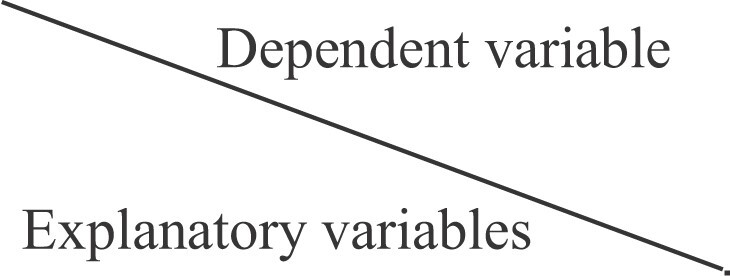				
	1	2	3	4
	Self-rated health	Physical pain	Depression	Injury
Part A				
Average Internet usage of the community	0.071^*^	0.055	0.998^***^	0.017
	(0.038)	(0.045)	(0.376)	(0.012)
Control variables	Yes	Yes	Yes	Yes
City FE	Yes	Yes	Yes	Yes
Observations	12 199	12 199	12 199	12 199
F statistic	20.291	12.068	8.380	7.503
Adjusted R^2^	0.194	0.123	0.087	0.041
Part B				
Internet usage	0.061^***^	0.020	0.393^***^	0.003
	(0.014)	(0.016)	(0.131)	(0.004)
Average Internet usage of the community	0.035	0.044	0.765^**^	0.016
	(0.039)	(0.046)	(0.389)	(0.012)
Control variables	Yes	Yes	Yes	Yes
City FE	Yes	Yes	Yes	Yes
Observations	12 199	12 199	12 199	12 199
F statistic value	20.299	11.984	8.388	7.501
Adjusted R^2^	0.196	0.124	0.088	0.041

Values in parentheses are the cluster robust standard errors. *p<0.1, **p<0.05, ***p<0.01. To save space, control variables and constants are not reported here. The control variables are the same as in Table 2.

As seen from part A of Table 5, when residents’ Internet use is not controlled for, the average community Internet use has a significant impact on self-rated health and depression indicators. After controlling for the Internet use of residents, as shown in part B, the average community Internet use has a significant impact on only the depression indicator and does not affect other indicators measuring the health of residents. Therefore, in general, the average Internet use of the community, excluding the household itself, is exogenous relative to the health of residents. Notably, the instrumental variables do not meet the exogeneity requirements for the depression indicator. Therefore, in the 2SLS regression analysis, the relationship between Internet use and this indicator is temporarily not considered.

The detailed assessment of the rationality of the instrumental variables shows that the instrumental variables selected in this article are suitable. This section uses the instrumental variables selected above for model estimation, and the results are presented in Table 6.

**Table 6.** 2SLS regression analysis

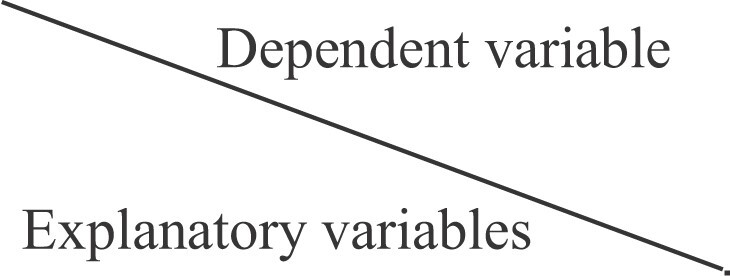				
	1	2	3	4
	Self-rated health	Physical pain	Depression	Injury
Internet usage	0.119^*^	0.093	1.682^***^	0.029
	(0.065)	(0.076)	(0.637)	(0.020)
Control variables	Yes	Yes	Yes	Yes
City FE	Yes	Yes	Yes	Yes
Observations	12 199	12 199	12 199	12 199
F statistical	20.334	11.987	8.167	7.122
Adjusted R^2^	0.195	0.122	0.079	0.038
DWH test	0.31	0.58	3.87^**^	1.89

Values in parentheses are the cluster robust standard errors. *p<0.1, **p<0.05, ***p<0.01. To save space, control variables and constants are not reported here. The control variables are the same as in Table 2.

First, the endogeneity of Internet use was tested. The Durbin–Wu–Hausman (DWH) test indicated failure to reject the null hypothesis for all of the health indicators, i.e. no endogeneity problems were detected. Therefore the endogeneity has been accounted for by the selection of control variables and the estimation results of the baseline regression are credible.

Although no endogeneity problem in Internet use is detected, the estimation results of the instrumental variables show that Internet use has a significant impact on residents’ self-rated health, which indicates that the impact of Internet use on residents’ health is robust.

In addition, when testing the correlation between instrumental variables and endogenous variables, to obtain the F statistic, the endogenous variable of Internet use is reprocessed. ‘Not surfing the Internet’ is assigned a value of 1, ‘only surfing the Internet with computers (including tablets)’ and ‘only using mobile phones’ are assigned a value of 2 and ‘surfing the Internet with both computers and mobile phones’ is assigned a value of 3. Therefore Internet use can be treated as a continuous value variable. Second, due to the difficulty of directly using the instrumental variable method to estimate the ordered probit model, this article adopts linear 2SLS, referring to the method of Zhou and Sun.^11^ Following the treatment specified above, the extent of the impact of Internet use on residents’ health in the basic model and the instrumental variable model are no longer comparable; however, the focus of this article is the nature of the impact, and in this regard the results are consistent.

In summary, the estimation results of the baseline regression and 2SLS regression show that Internet use has a significant positive impact on the health of residents in China.

## Further analysis

### Heterogeneity analysis

Health capital usually depreciates with an increase in age, while education is beneficial to reduce the depreciation rate of health capital.^37^ However, due to the influence of the traditional fertility concept and family resource constraints, women receive less human capital investment than men,^49^ resulting in women's worse health than men. Since residents can improve their health through the Internet, this part attempts to further analyse whether the impact of the Internet on residents’ health is different for age, education and gender, in particular, whether the Internet is conducive to improving the health of older, less educated and female residents.

In order to investigate whether there is heterogeneity in the impact of the Internet on residents’ health, this article constructs the cross terms of Internet and variables such as gender, education and age. Considering the possible collinearity problem, this article subtracts the mean values of Internet, gender, education and age, respectively. The results are shown in Table 7.

**Table 7.** Heterogeneity analysis

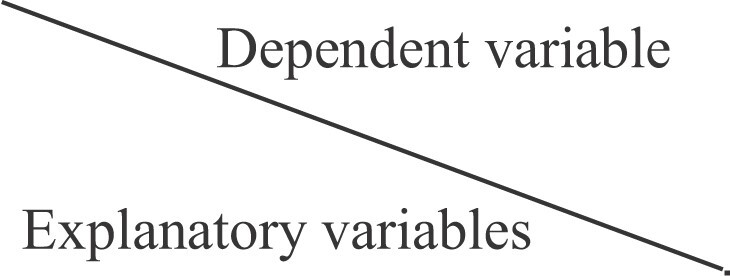				
	1	2	3	4
	Self-rated health	Physical pain	Depression	Injury
Part A: Gender				
Internet usage	0.138^***^	0.062^**^	0.645^***^	0.071^*^
	(0.029)	(0.029)	(0.233)	(0.043)
Male	0.128^***^	0.227^***^	1.787^***^	0.107^**^
	(0.026)	(0.028)	(0.194)	(0.045)
Male × Internet usage	−0.039	−0.071^*^	−0.764^**^	−0.156^**^
	(0.040)	(0.042)	(0.310)	(0.068)
Control variables	Yes	Yes	Yes	Yes
City FE	Yes	Yes	Yes	Yes
Observations	12 199	12 199	12 199	12 199
Wald/F statistical	2652.91	1658.63	8.353	516.27
Pseudo-R^2^/adjusted R^2^	0.086	0.054	0.088	0.072
Part B: Education				
Internet usage	0.115^***^	0.044	0.598^**^	0.063
	(0.031)	(0.030)	(0.242)	(0.045)
Education	0.008^**^	0.017^***^	0.120^***^	0.008
	(0.004)	(0.004)	(0.028)	(0.006)
Education × Internet usage	−0.015^**^	−0.012^*^	−0.031	−0.009
	(0.006)	(0.006)	(0.054)	(0.010)
Control variables	Yes	Yes	Yes	Yes
City FE	Yes	Yes	Yes	Yes
Observations	12 199	12 199	12 199	12 199
Wald/F statistical	2654.23	1660.47	8.357	507.76
Pseudo-R^2^/adjusted R^2^	0.086	0.054	0.087	0.072
Part C: Age				
Internet usage	0.123^***^	0.057^*^	0.593^**^	0.071
	(0.030)	(0.030)	(0.236)	(0.045)
Age	−0.027^***^	−0.020^***^	−0.033^***^	−0.007^***^
	(0.001)	(0.001)	(0.009)	(0.002)
Age × Internet usage	0.004^*^	0.001	0.013	0.002
	(0.002)	(0.002)	(0.016)	(0.003)
Control variables	Yes	Yes	Yes	Yes
City FE	Yes	Yes	Yes	Yes
Observations	12 199	12 199	12 199	12 199
Wald/F statistical	2652.34	1653.28	8.348	503.44
Pseudo-R^2^/adjusted R^2^	0.086	0.054	0.087	0.072

Values in parentheses are the cluster robust standard errors. *p<0.1, **p<0.05, ***p<0.01. To save space, control variables and constants are not reported here. The control variables are the same as in Table 2.

It can be seen from part A in Table 7 that the coefficient of gender is significantly positive, which indicates that men are healthier than women. However, in columns 2–4, the coefficient of the cross item of Internet and gender is significantly negative, indicating that compared with men, women can improve their health by using the Internet. Based on the above results, we can find that the Internet can reduce the health differences between the genders.

It can be seen from part B of Table 7 that the coefficient of education in columns 1–3 is significantly positive, indicating that with the improvement of education level, the health of residents can be improved. However, the coefficients of the cross items of Internet and education in columns 1 and 2 are significantly negative, indicating that compared with the residents with a higher education level, the residents with a lower education level can improve their health by using the Internet. Based on the above results, we can find that the Internet can reduce the health differences among residents with different educational backgrounds.

It can be seen from part C in Table 7 that the coefficient of age is significantly negative, which indicates that the health of residents is getting worse with an increase in age. However, the coefficient of the cross item of Internet and age in column 1 is significantly positive, indicating that compared with young people, the elderly can improve their health by using the Internet. Based on the above results, we find that the Internet can reduce the health differences among different ages. It is important to note that the cross coefficient between Internet and age is only significant in column 1, which is not robust enough, so further research is needed in the future.

### Influential mechanism analysis

At present, the mechanism of how Internet use affects the health of residents remains unclear.^8,9^ The existing literature on the possible theoretical mechanism of the impact of Internet use on the health of residents finds that Internet use can improve health by relaxing budget constraints and improving health production efficiency, but there are few empirical tests.

Therefore this article explores the transmission mechanism of how Internet use affects the health of residents from the perspective of employment performance. In view of the availability of data, income, job prestige and the autonomy of work content, work progress and work intensity are used as measures of employment performance to take into account the intermediary effects of budget constraints and healthy production efficiency. Income is expressed as the annual gross income of residents and is logarithmically transformed after adding one. According to Li's^50^ classification of occupational prestige, this part divides work types into five categories from low to high occupational prestige: 1 for agricultural, forestry, animal husbandry, fishery and water conservancy production personnel; 2 for commercial, service industry personnel and production and transportation workers; 3 for clerks; 4 for professional and technical personnel; and 5 for the person in charge of the unit. Work autonomy is defined as 1 for control by others, 2 for partial self-control and 3 for self-control. The above data were obtained from 2016 CLDS data.

The mediating effect model in this article is mainly a non-linear model and the Karlson–Holm–Breen (KHB) method^51^ can be used for mediating effect analysis of linear and non-linear models, as well as multidimensional mediating variables. Therefore this article uses the KHB method to test whether employment performance is the channel of Internet use affecting residents’ health.

**Figure 1.**
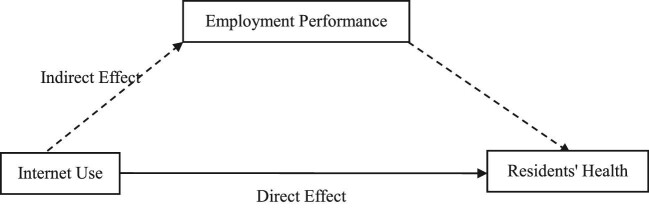
Influential mechanism.

Figure 1 shows the specific mechanism of Internet use affecting residents’ health. According to Figure 1, the impact of Internet use on residents’ health through the employment performance path is called an indirect effect and the impact of Internet use on residents’ health without any path is called a direct effect. The sum of the indirect effect and direct effect is the total effect of Internet use on residents’ health.

The results of the KHB method are reported in Table 8. In terms of the total effect, Internet use can improve self-rated health and reduce physical pain, depression and the probability of injury of residents. In terms of the direct effect, Internet use has a significant impact on residents’ self-rated health, physical pain and depression, but it does not affect the injury of residents. In terms of the indirect effect, the mediating variables have positive and significant effects on different health indicators of residents, indicating that employment performance plays a mediating role in the process of Internet use improving residents’ health. At the same time, comparing the direct effect with the total effect, it was found that in the self-rated health, physical pain and depression model, the direct effect is less than the total effect, while in the injury model, the direct effect is not only less than the total effect, but is also not significant, indicating that Internet use can partially improve residents’ self-rated health and reduce physical pain and depression through employment performance. However, the impact of Internet use on residents’ injuries is completely realized through employment performance.

**Table 8.** Mediating effect test based on KHB method

	1	2	3	4
Variables	Self-rated health	Physical pain	Depression	Injury
Total effect	0.140^***^	0.065^***^	0.637^***^	0.078^*^
	(0.029)	(0.029)	(0.232)	(0.043)
Direct effect	0.125^***^	0.053^*^	0.561^***^	0.066
	(0.029)	(0.029)	(0.232)	(0.042)
Indirect effect	0.015^***^	0.012^***^	0.077^***^	0.011^***^
	(0.003)	(0.003)	(0.019)	(0.004)
Proportion of indirect effects (%)	10.46	18.37	12.05	14.63
Income share	76.85	40.82	62.89	52.11
Proportion of job prestige	37.40	58.72	37.48	45.86
Proportion of work content autonomy	−6.88	−7.47	17.05	40.72
Proportion of work progress autonomy	14.76	17.45	−4.19	3.46
Proportion of work intensity and autonomy	−22.13	−9.53	−13.23	−42.15

Values in parentheses are the cluster robust standard errors. *p<0.1, **p<0.05, ***p<0.01. To save space, control variables and constants are not reported here. The control variables are the same as in Table 2.

From the perspective of the role of different mediating variables, different mediating variables play different roles. In different health models the mediating role of income fluctuated between 40.82% and 76.85% and that of work type fluctuated between 37.40% and 58.72%. The mediating role of work autonomy, such as work content, work progress and work intensity, was relatively small. Only in the injury model, the proportion of work content autonomy reached 40.72% and the proportion of work intensity autonomy was −42.15%. This shows that, on the whole, employment performance expressed by income and job type plays a major mediating role in the process of Internet use improving residents’ health, while employment performance expressed by work autonomy such as work content, work progress and work intensity plays a secondary mediating role in the process of Internet use improving residents’ health.

## Discussion and conclusions

With the rapid development of information technology, the Internet has become an important information infrastructure in the world, which plays a huge role in the healthy development of a country's economy and society. Along with it, academic research on the Internet has become increasingly extensive and in-depth. This article mainly examines the impact of Internet use on the health of Chinese residents and the mechanism of employment performance in the process. The results show that Internet use has a significant positive impact on the health of Chinese residents. Specifically, compared with not using the Internet, Internet use decreased the probability of residents’ self-rated health being classified as very unhealthy, relatively unhealthy and fair by 0.3%, 2.1% and 2.3%, respectively, and increased the probability of self-rated health being classified as relatively healthy and very healthy by 1.2% and 3.5%, respectively. The probability of always, often, sometimes and rarely experiencing physical pain decreased by 0.2%, 1.0%, 1.0% and 0.2%, respectively, and the probability of experiencing no pain increased by 2.3%. The degree of depression improved by approximately 0.644 and the occurrence of injuries decreased by 1.3%. Furthermore, the results in this article remain robust after selecting appropriate instrumental variables to address potential endogeneity. Heterogeneity analysis found that the Internet is conducive to improving the health of older, less educated and female residents. Considering that youth, higher education level and male residents have better health, the results of the heterogeneity analysis in this article suggest that the Internet can narrow the health gap of residents with different ages, education levels and genders, thus promoting health equality. The analysis of the impact mechanism shows that Internet use can improve the employment performance of Chinese residents, including higher income and higher occupational prestige, and then promotes health improvement. At the same time, this article points out that job autonomy, as a part of employment performance, does not play a significant mediating role after controlling income and occupational prestige.

Previous studies have shown that the elderly can easily communicate with the outside world with the help of the Internet, which is conducive to alleviating loneliness and improving their health.^10^ Although the research object of this article is the labour force, it also finds that the Internet can improve the health of residents. Different from the above research, this article analyses the specific mechanism of the Internet to improve residents’ health from the perspective of employment performance. The results show that with the help of the Internet, residents can get jobs with higher occupational prestige and higher income,^6^ which is conducive to improvement of their health. A job with higher occupational prestige means a better working environment, which is conducive to avoiding health damage caused by frequent exposure to hazardous substances.^52^ In addition, work is an important indicator to measure social status and work with higher occupational prestige also means higher social status, which is conducive to accumulating more social capital,^53^ so as to get more emotional support and help, and then improve health.^54^ Moreover, the increase of income is conducive to relaxing budget constraints faced in health investment, so as to directly improve health,^2^ such as regular physical examinations, leisure tourism, fitness exercise and so on.

Based on the research of different groups, this article finds that the Internet is conducive to improving the health of older, less educated and female residents, so as to narrow the health differences among residents of different ages, education levels and gender, and promote health equality. Previous studies have shown that compared with older, less educated and female residents, young, more educated and male residents have better employment performance.^55^ Therefore it is difficult to explain this phenomenon through the employment performance mechanism expressed by income and occupational prestige. Previous studies on the elderly have found that they can easily achieve the purpose of communication and exchange with others by virtue of the information transmission function of the Internet, which is conducive to obtaining social support and reducing loneliness, and ultimately promoting health improvement.^10^ With the help of the information transmission function of the Internet, older, less educated and female residents can achieve the purpose of health improvement through social communication. Xue et al.^56^ found that social capital can not only directly improve health, but can also indirectly improve health through mutual support, trust and social interaction to alleviate the negative effects of stress. This suggests that the information transmission function may be an important mechanism for the Internet to improve the health of older, less educated and female residents. In the future, with the help of abundant relevant data, this mechanism can be tested in detail to deepen the understanding of the relationship between the Internet and health.

Although previous studies on adolescents have found that using the Internet not only does not improve health, but also leads to health deterioration,^14^ this is mainly due to the poor self-control ability of adolescents. Compared with adults, adolescents are still in the growth stage and their minds are not mature. Facing the rich entertainment resources provided by the Internet, it is easy for them to become addicted, leading to health deterioration.^15^ For example, Billari et al.^16^ found that long-term use of the Internet can lead to decreased sleep satisfaction and insufficient sleep. Sleep disorders are prone to cause depression and damage health.^17^

Although this article discusses the relationship between Internet use and the health of Chinese residents and its impact mechanism and provides new findings, the limited availability of data has led to deficiencies that must be addressed. First, this article discusses only the dimension of whether to use the Internet and fails to explore the relationships between Internet use time and different uses of the Internet and health. Second, in terms of the specific mechanism of how Internet use affects health, this article focuses on only the intermediary effect of employment performance and fails to conduct an extensive in-depth analysis of other dimensions of health production efficiency. Even employment performance itself includes many dimensions. This article analyses only the dimensions of occupational prestige, work autonomy and income quantity and ignores other dimensions such as work pressure and income quality. Third, although the analysis of the impact mechanism shows that Chinese residents can improve their health by using the Internet to enhance their employment performance, the empirical results show that the indirect effect accounts for <20% of the total effect (as shown in Table 8), which means that employment performance plays a very limited role in the process of improving Chinese residents’ health through Internet use.^57^ In the future, we need to further explore the specific mechanism of Internet use to improve residents’ health. Finally, it should be noted that although this article attempts to deal with the possible endogeneity, it is difficult to completely solve the endogeneity problem caused by the time trend term, and city-specific time trends used cross-sectional data. Therefore an important contribution of this article is providing a possible direction for further research. In the future, the above problems must be addressed with more abundant data.

On the basis of the above analysis, this article proposes the following policy recommendations to promote the healthy development of China's economy and society.

First, China should vigorously develop the Internet, especially in rural areas. The results of this study show that Internet use can improve the health of residents, which is conducive to expanding approaches used to improve the health of residents and reducing the pressure on medical and health service systems. However, according to the 44th Statistical Report on Internet Development in China, the Internet penetration rate in China is 61.2%, far lower than the 81% in developed countries. Therefore development of the Internet is an important foundation for the healthy development of China's economy.

Second, the government and public welfare organizations should conduct public welfare training lectures on Internet use methods to improve Internet use skills. According to the data from the 44th Statistical Report on Internet Development in China, non-Internet users are concentrated mainly in rural areas and a lack of skills and limited education are the main reasons for not using the Internet. With limited access to medical resources in rural areas, the lack of Internet skills further limits the ability of rural residents to use the Internet to improve their health. Therefore an increase in non-profit Internet skills training would help to increase the number of Internet users and enhance the value of the Internet.

Third, we should appropriately guide use of the Internet, giving full play to its positive role and reducing its negative impact on the health of residents, especially minors with poor self-control. According to the 44th Statistical Report on Internet Development in China, the average weekly online time per user has been increasing since 2016, reaching 27.9 h in June 2019. Failure to control Internet use may have a negative impact on health.

## Data Availability

The data can be obtained from the following website: http://isg.sysu.edu.cn/node/353.
